# Association of Chronic Medical Conditions With Severe Outcomes Among Nonpregnant Adults 18–49 Years Old Hospitalized With Influenza, FluSurv-NET, 2011–2019

**DOI:** 10.1093/ofid/ofad599

**Published:** 2023-11-29

**Authors:** Efemona A Famati, Dawud Ujamaa, Alissa O’Halloran, Pam Daily Kirley, Shua J Chai, Isaac Armistead, Nisha B Alden, Kimberly Yousey-Hindes, Kyle P Openo, Patricia A Ryan, Maya L Monroe, Anna Falkowski, Sue Kim, Ruth Lynfield, Melissa McMahon, Kathy M Angeles, Sarah A Khanlian, Nancy L Spina, Nancy M Bennett, Maria A Gaitán, Eli Shiltz, Krista Lung, Ann Thomas, H Keipp Talbot, William Schaffner, Andrea George, Holly Staten, Catherine H Bozio, Shikha Garg

**Affiliations:** Influenza Division, Centers for Disease Control and Prevention, Atlanta, Georgia, USA; University of Minnesota Medical School, Minneapolis, Minnesota, USA; Influenza Division, Centers for Disease Control and Prevention, Atlanta, Georgia, USA; General Dynamics Information Technology, Falls Church, Virginia, USA; Influenza Division, Centers for Disease Control and Prevention, Atlanta, Georgia, USA; California Emerging Infections Program, Oakland, California, USA; California Emerging Infections Program, Oakland, California, USA; Career Epidemiology Field Officer Program, Centers for Disease Control and Prevention, Atlanta, Georgia, USA; Colorado Department of Public Health and Environment, Denver, Colorado, USA; Colorado Department of Public Health and Environment, Denver, Colorado, USA; Emerging Infections Program, Yale University School of Public Health, New Haven, Connecticut, USA; Georgia Emerging Infections Program, Atlanta, Georgia, USA; Atlanta Veterans Affairs Medical Center, Atlanta, Georgia, USA; Emory University School of Medicine, Atlanta, Georgia, USA; Maryland Department of Health, Baltimore, Maryland, USA; Maryland Department of Health, Baltimore, Maryland, USA; Michigan Department of Health and Human Services, Lansing, Michigan, USA; Michigan Department of Health and Human Services, Lansing, Michigan, USA; Minnesota Department of Health, St.Paul, Minnesota, USA; Minnesota Department of Health, St.Paul, Minnesota, USA; New Mexico Emerging Infections Program, Albuquerque, New Mexico, USA; New Mexico Emerging Infections Program, Albuquerque, New Mexico, USA; NewYork State Department of Health, Albany, New York, USA; University of Rochester School of Medicine and Dentistry, Rochester, NewYork, USA; University of Rochester School of Medicine and Dentistry, Rochester, NewYork, USA; Ohio Department of Health, Columbus, Ohio, USA; Ohio Department of Health, Columbus, Ohio, USA; Oregon Health Authority, Portland, Oregon, USA; Vanderbilt University School of Medicine, Nashville, Tennessee, USA; Vanderbilt University School of Medicine, Nashville, Tennessee, USA; Salt Lake County Health Department, Salt Lake City, Utah, USA; Salt Lake County Health Department, Salt Lake City, Utah, USA; Influenza Division, Centers for Disease Control and Prevention, Atlanta, Georgia, USA; Influenza Division, Centers for Disease Control and Prevention, Atlanta, Georgia, USA

**Keywords:** adults, chronic conditions, hospitalizations, influenza, severe

## Abstract

**Background:**

Older age and chronic conditions are associated with severe influenza outcomes; however, data are only comprehensively available for adults ≥65 years old. Using data from the Influenza Hospitalization Surveillance Network (FluSurv-NET), we identified characteristics associated with severe outcomes in adults 18–49 years old hospitalized with influenza.

**Methods:**

We included FluSurv-NET data from nonpregnant adults 18–49 years old hospitalized with laboratory-confirmed influenza during the 2011–2012 through 2018–2019 seasons. We used bivariate and multivariable logistic regression to determine associations between select characteristics and severe outcomes including intensive care unit (ICU) admission, invasive mechanical ventilation (IMV), and in-hospital death.

**Results:**

A total of 16 140 patients aged 18–49 years and hospitalized with influenza were included in the analysis; the median age was 39 years, and 26% received current-season influenza vaccine before hospitalization. Obesity, asthma, and diabetes mellitus were the most common chronic conditions. Conditions associated with a significantly increased risk of severe outcomes included age group 30–39 or 40–49 years (IMV, age group 30–39 years: adjusted odds ratio [aOR], 1.25; IMV, age group 40–49 years: aOR, 1.36; death, age group 30–39 years: aOR, 1.28; death, age group 40–49 years: aOR, 1.69), being unvaccinated (ICU: aOR, 1.18; IMV: aOR, 1.25; death: aOR, 1.48), and having chronic conditions including extreme obesity and chronic lung, cardiovascular, metabolic, neurologic, or liver diseases (ICU: range aOR, 1.22–1.56; IMV: range aOR, 1.17–1.54; death: range aOR, 1.43–2.36).

**Conclusions:**

To reduce the morbidity and mortality associated with influenza among adults aged 18–49 years, health care providers should strongly encourage receipt of annual influenza vaccine and lifestyle/behavioral modifications, particularly among those with chronic medical conditions.

Influenza virus infections resulted in an estimated 140 000–710 000 hospitalizations and 12 000–52 000 deaths annually in the United States from 2010 to 2020 [1]. In addition to causing substantial annual morbidity and mortality, influenza imposes significant costs on society including economic losses from direct medical costs at $10.4 billion annually and projected lost earnings from illness or loss of life ($16.3 billion annually) [2].

Factors that have been associated with a higher risk of severe influenza-associated outcomes among adults include age ≥65 years and chronic underlying conditions such as cardiac, lung, or metabolic disease [3–10]. In addition, higher rates of hospitalization and intensive care unit (ICU) admission have been observed in certain ethnic and racial groups including non-Hispanic Black (NH-Black), Hispanic or Latino, and American Indian or Alaska Native people compared with their Caucasian counterparts [11–13]. Although evidence suggests that certain groups experience worse outcomes when hospitalized with influenza, most studies have focused on adults aged ≥65 years and children aged <5 years, as these groups historically have the highest disease burden [8]. There is a lack of well-documented information regarding characteristics and outcomes of younger adults hospitalized with influenza. Using a large and geographically diverse influenza hospitalization surveillance network, this study aimed to provide further information on the association of underlying medical conditions with severe outcomes among adults 18–49 years old hospitalized with influenza.

## METHODS

### Description of Surveillance

The Centers for Disease Control and Prevention (CDC)–sponsored Influenza Hospitalization Surveillance Network (FluSurv-NET) is a US population-based surveillance system that collects data on laboratory-confirmed influenza-associated hospitalizations from October 1st to April 30th of each influenza season [14]. During the 8 influenza seasons included in this analysis, 2011–2012 through 2018–2019, FluSurv-NET surveillance took place in selected counties in 15 states participating in the Emerging Infection Program (California, Colorado, Connecticut, Georgia, Maryland, Minnesota, New Mexico, New York, Oregon, and Tennessee) or the Influenza Hospitalization Surveillance Project (Iowa [IA; 2012–2013], Michigan, Ohio, Rhode Island [RI] [2011–2012 to 2012–2013], and Utah), with a catchment population of ∼29 million persons [15]. Hospitalized residents of the FluSurv-NET catchment area who had a positive influenza test (by molecular assay, rapid antigen testing, direct or indirect fluorescent antibody staining, viral culture, or a positive influenza test noted in the medical chart with an associated test date) during hospitalization or up to 14 days before hospital admission were included as cases. Surveillance staff reviewed multiple data sources including hospital and laboratory records, infection control practitioner logs, and reportable condition databases to identify FluSurv-NET cases.

This analysis included adults 18–49 years of age, hospitalized with influenza during the 2011–2012 through 2018–2019 influenza seasons. We excluded cases from IA and RI because these sites contributed small amounts of data (n = 188 cases) for 2 seasons or less. In contrast, all other sites provided data for each year within the analytic period (2011–2012 to 2018–2019). Cases with hospital onset (defined as positive influenza test >3 days after hospital admission) and persons who were pregnant at the time of hospital admission were also excluded. Trained surveillance staff used a standardized case report form to conduct medical chart abstractions on all identified cases to collect information on patient demographics and clinical characteristics, underlying condition categories (asthma/reactive airway disease, chronic lung disease, chronic metabolic disease, blood disorders, hemoglobinopathy, cardiovascular disease, neuromuscular/neurologic disorder, immunocompromised conditions, renal disease, liver disease, obesity) (Supplementary Figure 1), receipt of current season influenza vaccine, receipt of antiviral treatment, and outcomes (ICU admission, pneumonia as previously defined [16], invasive mechanical ventilation [IMV], and death from any cause) during an influenza-associated hospitalization. Receipt of current-season influenza vaccination was ascertained using up to 4 sources: hospital medical records, state immunization registries, outpatient provider records, and through interview of the patient or their proxy [17].

### Statistical Analysis

The frequencies of demographic and clinical characteristics and outcomes were calculated for nonpregnant adults 18–49 years of age hospitalized with influenza during the 2011–2012 through 2018–2019 influenza seasons. The distribution of underlying condition categories and receipt of current-season influenza vaccine were examined by age group, sex, race, and ethnicity. Pearson correlation coefficients were used to determine the correlation between underlying condition categories. Bivariate and multivariable logistic regression models were used to determine associations between select characteristics, determined a priori and/or found to be significant in bivariate analyses (age, sex, race and ethnicity, surveillance site, season, vaccination status, tobacco use, and the presence of underlying condition categories [asthma, chronic lung disease, chronic metabolic disease, cardiovascular disease, blood disorders, neurologic disease, immunosuppressive conditions, liver disease, and extreme obesity]) on the individual outcomes of ICU admission, community-acquired pneumonia, IMV, and death. A separate multivariable logistic regression model was also created to assess the independent association between the number of chronic medical conditions as a continuous variable and each severe outcome. Obesity was calculated based on body mass index (BMI), and if BMI was missing, by International Classification of Diseases discharge diagnosis codes (E66.0, E66.09, E66.1, E66.8, E66.9, E66.01, E66.2, or Z68.4) or by clinician documentation of obesity on a problem list. Obesity was defined as BMI ≥30 kg/m^2^, and extreme obesity was defined as BMI ≥40 kg/m^2^ [18]. *P* values were calculated using Pearson chi-square tests. Statistical significance was set at α = .05; all tests were 2-sided. Odds ratios and exact 95 CIs were calculated for each outcome by setting a reference group for each characteristic. Statistical analyses were performed in SAS, version 9.4 (SAS Institute).

The CDC determined that this activity met the requirement for public health surveillance; therefore, CDC Institutional Review Board (IRB) approval was not required. Sites participating in FluSurv-NET obtained human subjects and ethics approvals from their respective state and local health department and academic partner IRBs as needed.

## RESULTS

### Characteristics of Analytic Population

**Figure 1.**
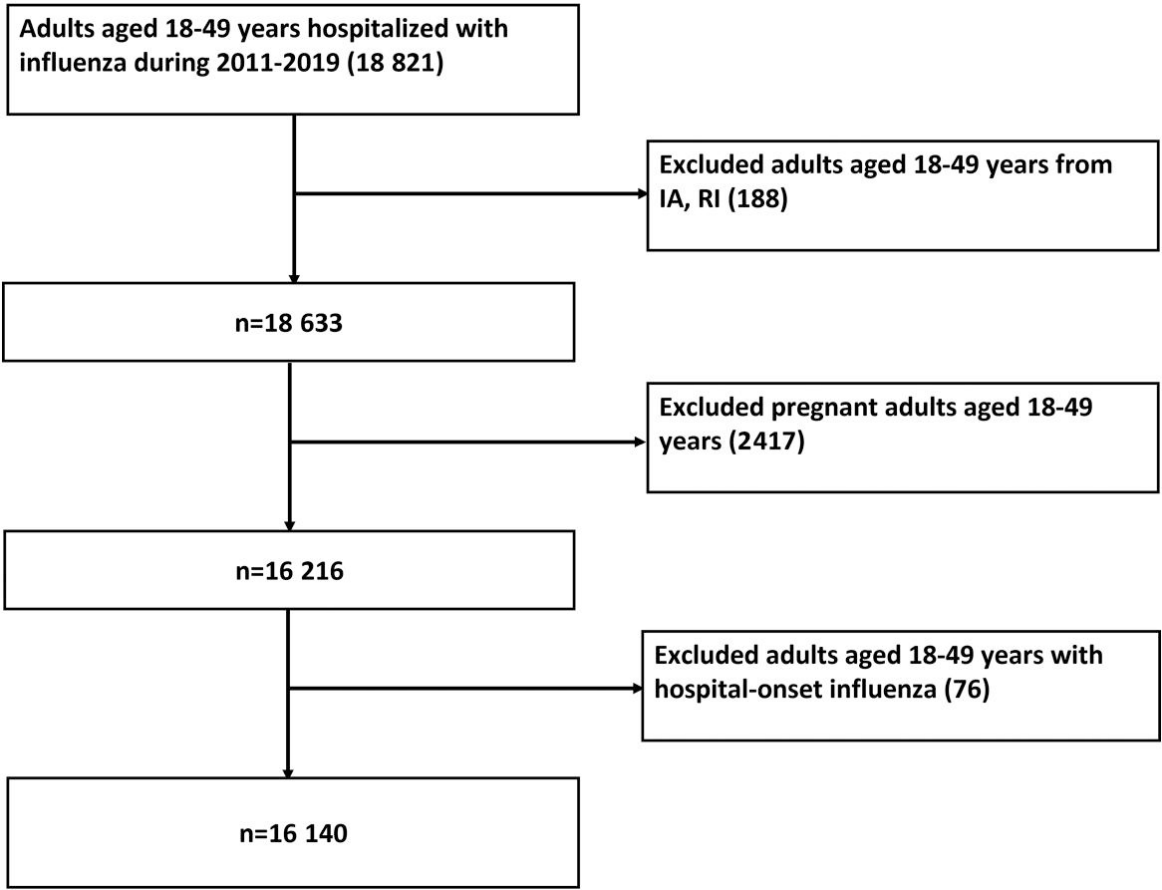
FluSurv-NET study population, 2011–2012 to 2018–2019.

This repeated cross-sectional analysis began with 18 821 adults 18–49 years old hospitalized with influenza during the 2011–2012 through 2018–2019 influenza seasons. After excluding cases from IA and RI (n = 188), cases with hospital-onset influenza as previously defined (n = 76) [17], and persons who were pregnant at the time of hospital admission (n = 2417), we included a total of 16 140 patients in the analysis (Figure 1).

The median age of included patients (interquartile range [IQR]) was 39 (30–45) years, and 52% were female (Table 1); 44.1% of patients were non-Hispanic White (NH-White), 30.5% were NH-Black, 12.3% were Hispanic, 3.8% were non-Hispanic Asian or Pacific Islander, 1.1% were non-Hispanic American Indian or Alaska Native, <0.5% were of multiple races and ethnicities, and 7.6% had unknown or missing race and ethnicity. The majority of patients (58.6%) were unvaccinated, 26.1% were vaccinated, and 15.3% had unknown vaccination status. Vaccination status did not vary significantly by age, but there were differences by race and ethnicity: NH-White (28.5%), NH-Black (24.6%), Hispanic (22.9%), other races (24.9%) (Supplementary Figure 2). Among a subset of 6123 patients with information on insurance status, 51.8% had Medicare or Medicaid and 41.7% had private insurance (Table 1). A total of 33.3% of patients were current smokers. Most patients (93.7%) had febrile or respiratory symptoms at admission. Among 15 110 patients with a date of respiratory symptom onset, the median number of days (IQR) from symptom onset to admission was 3 (1–4) days.

**Table 1.** Characteristics, Interventions, and Outcomes of Nonpregnant Adults Aged 18–49 Years Hospitalized With Influenza, FluSurv-NET, 2011–2012 Through 2018–2019 (n = 16 140)

	No. (%)
Total	16 140
Age, y	
Age, median (IQR), y	39 (30–45)
18–29 y	3788 (23.5)
30–39 y	4706 (29.2
40–49 y	7646 (47.4)
Sex	
Male	7746 (48.0)
Female	8394 (52.0)
Race/ethnicity	
White, NH	7123 (44.1)
Black, NH	4930 (30.5)
Hispanic	1989 (12.3)
Other^a^	2098 (13.0)
Insurance status^b^	
Medicare, Medicaid	3174 (51.8)
Private	2556 (41.7)
Uninsured	550 (9.0)
Unknown	265 (4.3)
Other^c^	171 (2.8)
Residence at admission	
Private residence	14 726 (91.2)
Other^d^	1414 (8.8)
Discharge location	
Private residence	13 379 (82.9)
Other^e^	2761 (17.1)
Influenza season	
2011–2012	433 (2.7)
2012–2013	1605 (9.9)
2013–2014	2368 (14.7)
2014–2015	1908 (11.8)
2015–2016	1801 (11.2)
2016–2017	1902 (11.8)
2017–2018	3337 (20.7)
2018–2019	2786 (17.3)
Influenza type^f^	
Influenza A	13 140 (81.4)
Influenza B	2891 (17.9)
Influenza vaccination status	
Yes	4220 (26.1)
No	9453 (58.6)
Unknown	2467 (15.3)
Antiviral treatment	14 100 (87.4)
18–29 y age group	3271 (86.4)
30–39 y age group	4137 (87.9)
40–49 y age group	6692 (87.5)
Any symptoms at admission^g^	15 919 (98.6)
Any febrile or respiratory symptom^h^	15 120 (93.7)
Congestion or runny nose	3658 (22.7)
Fever or chills	9997 (61.9)
Cough	9295 (57.6)
Shortness of breath	6571 (40.7)
Tobacco use	
Current smoker	5370 (33.3)
Former smoker	1948 (12.1)
No/unknown smoker^i^	8822 (54.7)
Underlying medical conditions^j^	
Obesity or extreme obesity^k^	7083 (44.7)
Extreme obesity only	2653 (16.7)
Asthma	5157 (32.4)
Chronic metabolic disease	4262 (26.8)
Diabetes	3229 (20.3)
Thyroid dysfunction	1194 (7.5)
Immunocompromised condition	3101 (19.5)
HIV infection	740 (4.7)
Cardiovascular disease	2749 (17.3)
Coronary artery disease	527 (3.3)
Neurologic disorder	2743 (17.3)
Chronic lung disease (excluding asthma)	2093 (13.2)
Emphysema or COPD	1188 (7.5)
Renal disease	1705 (10.7)
Blood disorders/hemoglobinopathy	904 (5.7)
Sickle cell disease	325 (2.0)
Liver disease	747 (4.7)
No underlying medical conditions	2490 (15.7)
No. of underlying medical conditions	
0	2490 (15.7)
1	1907 (12.0)
2	2676 (16.8)
≥3	8821 (55.5)
Outcome^l^	
Length of stay, median (IQR), d	3 (2–5)
ICU admission	3129 (19.5)
Pneumonia	3893 (24.1)
Invasive mechanical ventilation	1340 (8.4)
Died during hospitalization	269 (1.7)

Abbreviations: BMI, body mass index; COPD, chronic obstructive pulmonary disease; ICD-10, *International Classification of Diseases, 10th Revision*; ICU, intensive care unit; ILI, influenza-like illness; IQR, interquartile range; LTACH, long-term acute care hospital; LTCF, long-term care facility; TCU, transitional care unit; URI, upper respiratory infection.

^a^Other includes American Indian/Alaska Native (n = 174), Asian Pacific Islander (n = 615), Multiracial (n = 63), unknown (n = 1234), or missing (n = 12).

^b^Insurance status data were only available for the 2017–2018 and 2018–2019 seasons (n = 6123), and categories are not mutually exclusive.

^c^Other insurance includes military, Indian Health Service, incarcerated; other insurance types entered as free text.

^d^Other residence at admission includes nursing home/skilled nursing home/hospice/extended care (n = 169), rehabilitation facility (n = 30), group home/retirement home (n = 263), assisted living/residential care (n = 57), homeless/shelter (n = 570), other/home with services (n = 104), psychiatric facility (n = 35), corrections facility (n = 50), LTACH/TCU (n = 7), other long term care facility (n = 2), alcohol/drug abuse treatment (n = 37), unknown (n = 57), missing (n = 33).

^e^Other discharge location includes nursing home/skilled nursing home/hospice/extended care (n = 253), rehabilitation facility (n = 215), group home/retirement home (n = 204), assisted living/residential care (n = 40), homeless/shelter (n = 421), other/home with services (n = 890), psychiatric facility (n = 66), corrections facility (n = 41), LTACH/TCU (n = 80), other LTCF (n = 48), alcohol/drug abuse treatment (n = 47), unknown (n = 132), missing (n = 324).

^f^For influenza type, the proportion of cases with influenza A&B was 77 (0.5), influenza A or B not distinguished was 26 (0.2), and unknown was 6 (0.0).

^g^Any symptoms at admission include acute respiratory illness, asthma and/or COPD exacerbation, pneumonia, other respiratory or cardiac conditions (seasons 2011–2012–2013–2014); chest pain, conjunctivitis/pink eye, diarrhea, headache, myalgia/muscle aches, nausea/vomiting, rash (seasons 2014–2015–2017–2018); altered mental status/confusion, congested/runny nose, cough, seizures, shortness of breath/respiratory distress, sore throat, wheezing, URI/ILI (seasons 2014–2015–2018–2019); fatigue/weakness (seasons 2015–2016–2017–2018); fever/chills (seasons 2012–2013–2018–2019); and other, nonrespiratory symptoms (seasons 2011–2012–2017–2018). One hundred eleven (0.7%) cases are missing data for all symptoms.

^h^Any febrile or respiratory symptoms include acute respiratory illness, asthma and/or COPD exacerbation, other respiratory or cardiac conditions (seasons 2011–2012–2013–2014); fever/chills (seasons 2012–2013–2018–2019); congested/runny nose, cough, shortness of breath/respiratory distress, sore throat, wheezing, URI/ILI (seasons 2014–2015–2018–2019).

^i^Data on underlying conditions missing for n = 246 (1.5%) cases.

^j^No/unknown smoker (tobacco) includes no/unknown (n = 8469) and missing (n = 353).

^k^Obesity is determined by calculated BMI (≥95th percentile), obesity or morbid obesity selected as an underlying medical condition, or ICD-10 code E66.0, E66.09, E66.1, E66.8, E66.9, E66.01, E66.2, or Z68.4 entered as a discharge diagnosis.

^l^Cases with unknown and missing values for outcome variables were excluded. For ICU admission, 34 (0.2%) cases were missing and 50 (0.3%) cases were unknown. For invasive mechanical ventilation, 43 (0.3%) cases were missing and 58 (0.4%) cases were unknown. For in-hospital death, 35 (0.2%) cases were missing and 18 (0.1%) cases were unknown. For pneumonia, no cases were missing or unknown. Pneumonia was defined as a combination of radiographic findings of bronchopneumonia, air space opacity, consolidation, lobar or interstitial infiltrate within 3 days of hospital admission, and either an ICD-10-coded discharge diagnosis of pneumonia or documentation of pneumonia on the hospital discharge summary.

**Figure 2.**
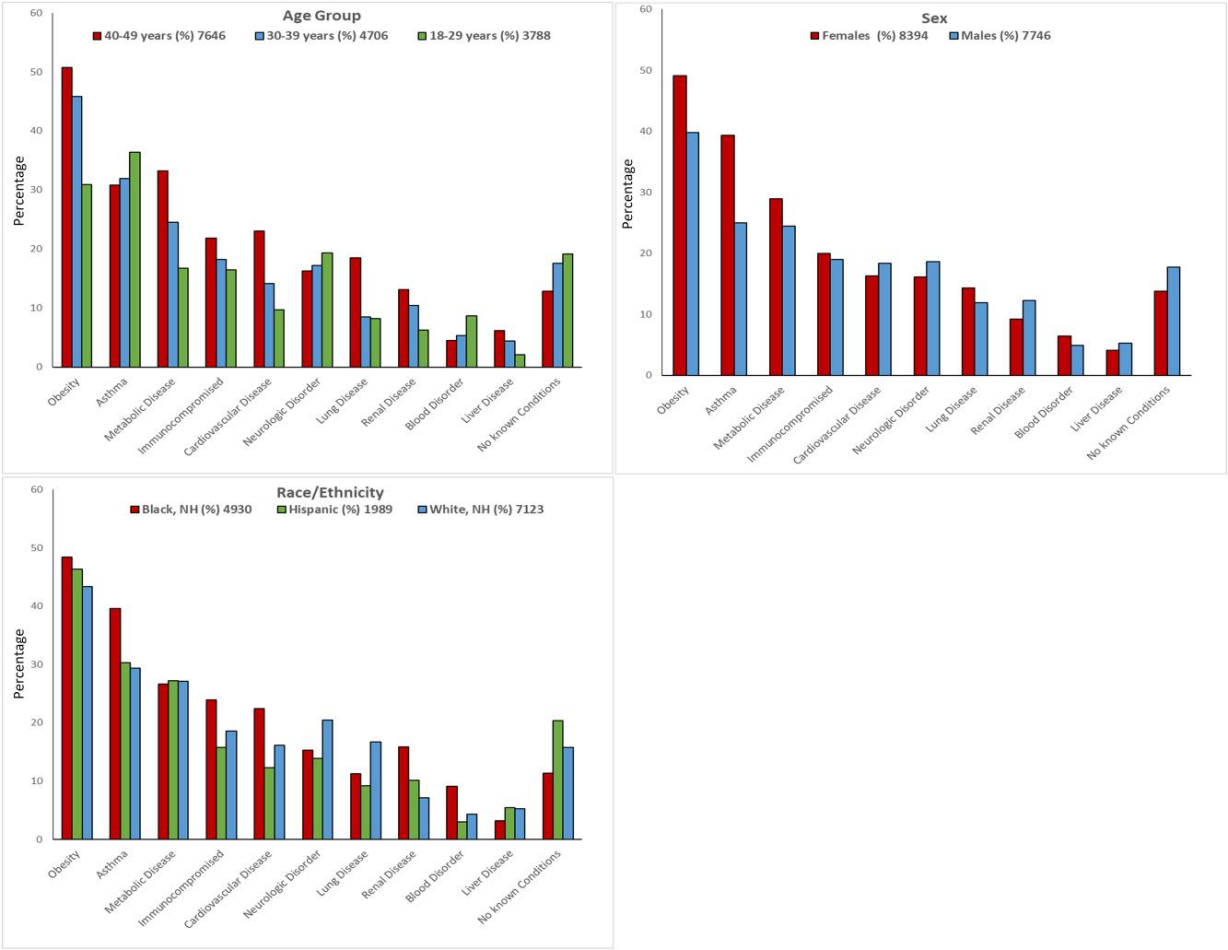
Distribution of underlying condition categories by age group, sex, and race/ethnicity, FluSurv-NET, 2011–2012 to 2018–2019.

Most (84.3%) patients had at least 1 underlying medical condition category; 15.7% had no underlying conditions present, 12.0% had 1, 16.8% had 2, and 55.5% had ≥3 condition categories. Obesity (including extreme obesity) was the most common condition (44.7%), followed by asthma (32.04%) and diabetes mellitus (20.3%) (Table 1). Underlying condition categories were not found to be highly correlated with each other (Supplementary Table 1). The distribution of underlying condition categories by age, sex, and race and ethnicity is displayed in Figure 2. Across age groups, 50.7% of patients aged 40–49 years, 45.8% aged 30–39 years, and 30.9% aged 18–29 years had obesity. By race and ethnicity, 43.3% of NH-White patients, 48.4% of NH-Black patients, and 46.3% of Hispanic patients had obesity. Asthma was the most common underlying medical condition among patients aged 18–29 years (36.4%) and the second most common condition among those aged 30–39 years (31.9%). A higher proportion of NH-Black patients had asthma (39.6%) compared with Hispanic (30.3%) and NH-White (29.4%) patients. Chronic metabolic disease (75% of which consisted of diabetes mellitus) was the second most common underlying condition (33.2%) among patients aged 40–49 years.

The majority of patients (87.4%) received antiviral therapy, with similar treatment coverage across age groups (Table 1). The median length of hospital stay (IQR) was 3 (2–5) days. A total of 3893 (24.1%) patients developed pneumonia, 3129 (19.5%) patients were admitted to the ICU, 1340 (8.4%) required IMV, and 269 (1.7%) died in the hospital. Bivariate associations between demographic and clinical characteristics and the outcomes of pneumonia, ICU admission, IMV, and death are displayed in Supplementary Table 2.

### Factors Associated With Severe Outcomes, Adjusted Analyses

**Figure 3.**
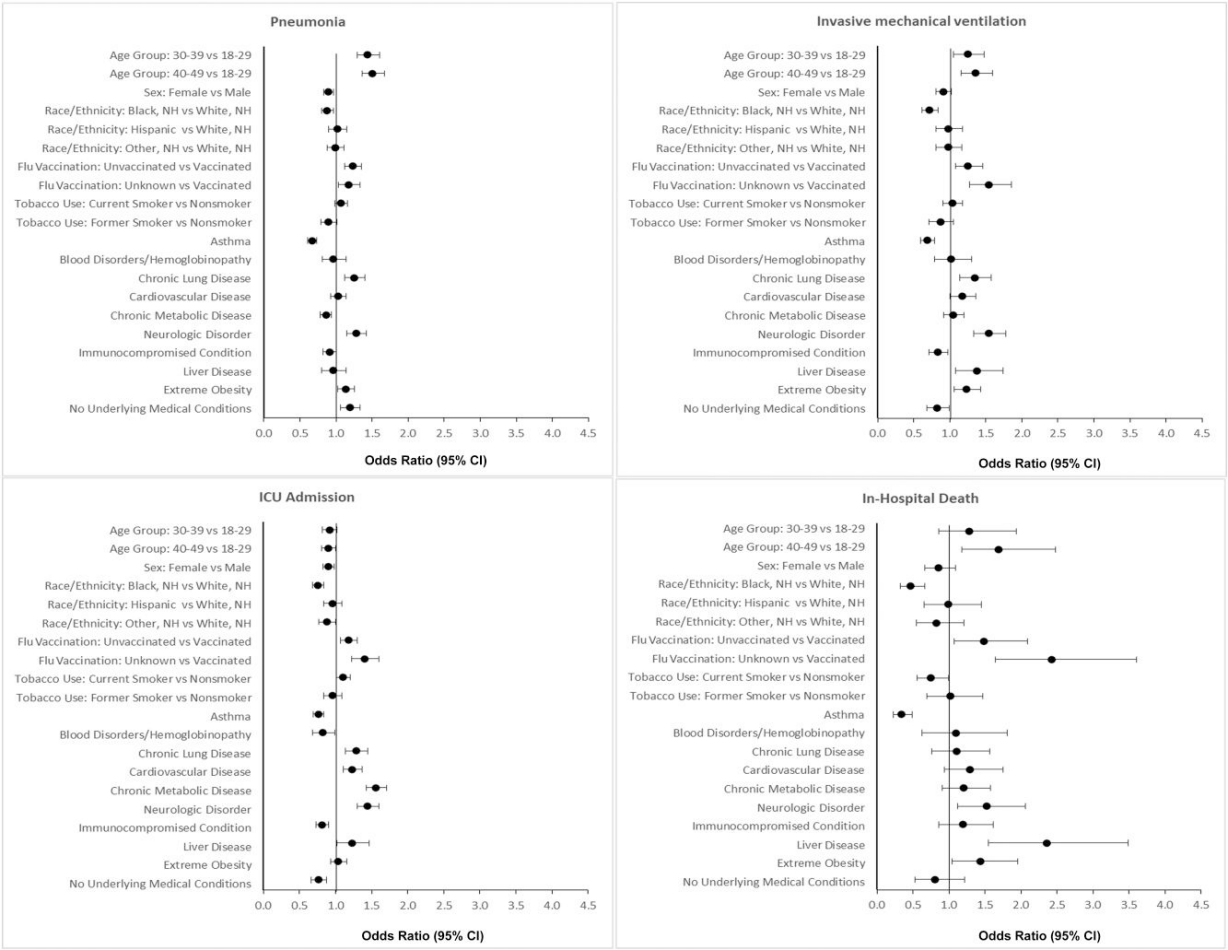
Factors associated with severe outcomes among adults aged 18–49 years hospitalized with influenza, FluSurv-NET, 2011–2012 to 2018–2019. Each model (pneumonia, ICU admission, invasive mechanical ventilation, and in-hospital death) was adjusted for surveillance site, influenza season, age, sex, race/ethnicity, influenza vaccination status, tobacco use, any vs no underlying condition, and the following underlying condition categories: asthma, blood disorders/hemoglobinopathy, chronic lung disease, cardiovascular disease, metabolic disease, neurologic disease, immunocompromising condition, liver disease, extreme obesity. Abbreviation: ICU, intensive care unit.

In multivariable analyses, patients aged 30–39 and 40–49 years old, respectively, were more likely to develop pneumonia (patients aged 30–39: adjusted odds ratio [aOR], 1.44; 95% CI, 1.29–1.61; patients aged 40–49: aOR, 1.51; 95% CI, 1.36–1.67) or require IMV (patients aged 30–39: aOR, 1.25; 95% CI, 1.05–1.48; patients aged 40–49: aOR, 1.36; 95% CI, 1.16–1.59), and patients aged 40–49 years were more likely to die in the hospital (aOR, 1.69; 95% CI, 1.18–2.48) compared with those aged 18–29 years (Figure 3; Supplementary Table 3). NH-Black patients had significantly lower odds of all severe outcomes compared with NH-White patients (pneumonia: aOR, 0.88; 95% CI, 0.9–0.97; ICU admission: aOR, 0.75; 95% CI, 0.68–0.84; IMV: aOR, 0.72; 95% CI, 0.62–0.84; in-hospital death: aOR, 0.47; 95% CI, 0.32–0.66). Unvaccinated patients had higher odds of all severe outcomes compared with vaccinated patients (pneumonia: aOR, 1.23; 95% CI, 1.12–1.36; ICU admission: aOR, 1.18; 95% CI, 1.07–1.3; IMV: aOR, 1.25; 95% CI, 1.08–1.45; in-hospital death: aOR, 1.48; 95% CI, 1.07–2.09). Several underlying condition categories, including chronic lung disease (pneumonia: aOR, 1.25; 95% CI, 1.12–1.41; ICU: aOR, 1.28; 95% CI, 1.14–1.44; IMV: aOR, 1.34; 95% CI, 1.14–1.58), cardiovascular disease (ICU: aOR, 1.23; 95% CI, 1.1–1.37; IMV: aOR, 1.17; 95% CI, 1.01–1.36), chronic metabolic disease (ICU: aOR, 1.56; 95% CI, 1.42–1.71), neurologic disease (pneumonia: aOR, 1.28; 95% CI, 1.15–1.42; ICU: aOR, 1.44; 95% CI, 1.3–1.6; IMV: aOR, 1.54; 95% CI, 1.33–1.78; death: aOR, 1.53; 95% CI, 1.12–2.06), liver disease (ICU: aOR, 1.22; 95% CI, 1.02–1.46; IMV: aOR, 1.38; 95% CI, 1.08–1.74; death: aOR, 2.36; 95% CI, 1.55–3.48), and extreme obesity (pneumonia: aOR, 1.13; 95% CI, 1.02–1.26; IMV: aOR, 1.23; 95% CI, 1.06–1.43; death: aOR, 1.43; 95% CI, 1.04–1.95) were associated with significantly higher odds of ≥1 severe outcome. Other underlying condition categories, including asthma, blood disorders, and immunocompromising conditions, were associated with lower odds of ≥1 severe outcome. Compared with patients with no underlying medical condition categories, patients with ≥3 underlying condition categories were more likely to be admitted to the ICU (aOR, 1.68; 95% CI, 1.48–1.9), require IMV (aOR, 1.45; 95% CI, 1.22–1.73), and die (aOR, 1.74; 95% CI, 1.2–2.6), but less likely to develop pneumonia (aOR, 0.76; 95% CI, 0.68–0.84) (Supplementary Table 4).

## DISCUSSION

Using data collected over 8 seasons from a large, population-based influenza hospitalization surveillance network, we found that among nonpregnant adults aged 18–49 years hospitalized with influenza, the median age was 39 years, most had ≥1 underlying medical condition category, and over half of patients had ≥3 underlying condition categories. Nearly a quarter of patients developed pneumonia, almost 20% required admission to the ICU, 8% received IMV, and almost 2% died in the hospital. This study found that the presence of certain underlying medical conditions, including chronic lung disease, cardiovascular disease, neurologic disorders, liver disease, and extreme obesity increased the odds of severe outcomes in this population. In addition, the odds of severe outcomes were substantially increased among patients with ≥3 chronic conditions compared with those with no chronic conditions.

People with chronic lung disease were more likely to be admitted to the ICU, require IMV, and develop pneumonia. Our findings are similar to prior studies that identified increased rates of severe outcomes in people with chronic lung disease [10, 19, 20]. A meta-analysis of severe influenza illness in 610 782 participants found that individuals with chronic lung disease had the highest rate of ICU admission and ventilatory support compared with people with other medical comorbidities [3]. Our data showed that people with asthma were less likely to experience severe outcomes compared with people without asthma. These findings are consistent with prior studies that have seen an increased hospitalization rate in asthmatics without an increase in severe outcomes [21, 22]. Similar findings were not observed in people with other chronic respiratory conditions. Coleman et al. noted that one possible explanation for the observed protective effect of asthma on people hospitalized with influenza is that asthma is not a major risk factor for death in the United States, although it is a common underlying condition [22]. Another explanation is that health care providers may have a lower threshold to admit people with asthma to the hospital; it is well documented that viral respiratory infections can exacerbate asthma [23]. Health care professionals may have a bias toward admitting people with asthma, even if they present with less severe disease, which would allow patients to receive treatment earlier in their disease course [5].

In our analysis, about 17% of patients had extreme obesity. Obesity conferred an increased risk of in-hospital death, ICU admission, and IMV. Obesity is a well-known risk factor for severe complications in people hospitalized with influenza. A prior study of people hospitalized with influenza during the influenza A/H1N1 pandemic found that patients with extreme obesity were twice as likely to be admitted to the ICU or die [24].

We found that unvaccinated patients were more likely to be admitted to the ICU, require IMV, die in-hospital, and develop pneumonia compared with vaccinated patients. Influenza vaccination is a major preventive strategy used each influenza season to reduce the incidence and severity of influenza. Only 26% of people in this study were vaccinated, which was lower than the average vaccination rates in the general population aged 18–49 during 2010–2022 (26.9%–38.4%) in the United States [25]. This suggests that vaccinated persons may have been less likely to be hospitalized with influenza during influenza seasons. Previous studies have shown that unvaccinated people hospitalized with influenza are more likely to develop severe outcomes compared with hospitalized, vaccinated patients who develop influenza [26–28]. One study found that significantly more unvaccinated people were admitted to the hospital or died and that vaccination reduced these rates by 23% in people aged 18 years and older [28]. These results coupled with our study suggest that influenza vaccine may attenuate influenza disease severity.

Our results highlight key areas where preventive measures can reduce the severity of influenza-associated hospitalizations. Underlying medical conditions such as obesity and chronic lung, cardiovascular, neurologic, and liver disease were associated with worse outcomes. The National Center for Chronic Disease Prevention and Health Promotion estimates that nearly 60% of US adults have a chronic health condition [29]. One study of 25 417 people conducted in 2018 found that almost 21% of adults 18–49 years old had ≥1 chronic medical condition [30]. Although our study was not designed to assess the impact of management and control of chronic conditions on risk of severe influenza-associated outcomes, prevention and control of chronic conditions in younger adults may reduce disease progression and the likelihood of severe complications of influenza [8]; future work in this area is needed.

Influenza vaccination is the most critical intervention to prevent and attenuate influenza disease. Younger adults are less likely to be vaccinated against influenza than older adults. One study from 2010–2011 to 2015–2016 of patients with severe laboratory-confirmed influenza hospitalizations found that influenza vaccine uptake was significantly higher in individuals aged ≥65 years compared with people aged 18–64 years (41.6% vs 8.9%) [28]. Although people aged ≥65 years are commonly considered higher risk for severe complications of influenza [31], our study shows that younger adults also experience severe influenza-associated outcomes, especially those with certain underlying medical conditions. Generally, this population consists of working-aged adults; therefore, hospitalization and death have a significant effect on US productivity. Messaging around the importance of influenza vaccination should be targeted toward the entire population, with specific consideration for younger adults.

Unexpectedly in our analysis, NH-Black people were less likely to develop pneumonia, be admitted to the ICU, require IMV, or die in the hospital compared with NH-White people. Hispanic people had similar odds of severe outcomes compared with their NH-White counterparts. Prior studies have found that NH-Black and Hispanic people were more likely to be hospitalized with influenza and experience severe outcomes [11, 13, 32]. In 1 FluSurv-NET analysis that examined population-based rates of influenza hospitalization, ICU admission, and in-hospital death by race and ethnicity, NH-Black people had the highest rates of ICU admission, followed by American Indian or Alaska Native and Hispanic persons, after adjusting for age [11]. One explanation for the discrepancy between our findings and those of prior studies is our population demographic. The individuals in our study were already hospitalized with influenza, and the findings may be confounded by biases related to access to care and admission practices. These biases may be better accounted for when performing analyses that start with the general nonhospitalized population. For example, NH-Black people in this analysis had a higher burden of several chronic conditions, such as asthma, compared with other racial and ethnic groups, which may have lowered the clinical threshold for admission and early intervention, even among patients presenting with less severe disease. Ultimately, we were unable to account for some of these potential complexities between race and ethnicity, types of underlying conditions, and resulting morbidity. Additional analyses are warranted to further explore these findings.

This study has several limitations. First, the FluSurv-NET surveillance catchment area covers ∼9% of the United States population; thus, our findings may not be nationally representative. Influenza testing in FluSurv-NET is clinician-driven or based on facility testing practices, and underascertainment of influenza-associated hospitalization may have occurred. We were unable to account for biases related to hospital admission practices. Admission-related practices may have explained several findings in our analysis including the observation that patients with asthma, blood disorders (including sickle cell anemia), and immunocompromising conditions had lower odds of severe outcomes compared with those without these conditions, potentially due to lower thresholds for admission of patients with these underlying conditions, even in the absence of severe disease [5, 33]. Data on race and ethnicity were missing or unknown for 7.7% of our study population, which could have led to misclassification bias. Finally, there were likely unmeasured confounders that we were unable to account for in this analysis, which may help to explain the unexpected findings, including the observation that NH-Black persons had lower odds of severe in-hospital outcomes compared with NH-White persons.

## CONCLUSIONS

Chronic medical conditions such as cardiovascular disease, chronic lung disease, and obesity are associated with more severe outcomes in adults aged 18–49 years hospitalized with influenza. These data support the importance of preventive medicine efforts in younger adults including annual influenza vaccination, particularly among those with chronic underlying conditions.

## Supplementary Material

ofad599_Supplementary_Data

## Supplementary Data

Supplementary materials are available at *Open Forum Infectious Diseases* online. Consisting of data provided by the authors to benefit the reader, the posted materials are not copyedited and are the sole responsibility of the authors, so questions or comments should be addressed to the corresponding author.

## Notes

***Patient consent.*** This study does not include factors necessitating patient consent.

***Financial support*.** This work was supported by the Centers for Disease Control and Prevention through an Emerging Infections Program cooperative agreement (grant CK17–1701) and through a Council of State and Territorial Epidemiologists cooperative agreement (grant NU38OT000297–02–00).
