# Supplementary material for: Association of Chronic Medical Conditions With Severe Outcomes Among Nonpregnant Adults 18–49 Years Old Hospitalized With Influenza, FluSurv-NET, 2011–2019
Source: Open Forum Infect Dis. 2023 Nov 29;10(12):ofad599. doi: 10.1093/ofid/ofad599 (PMC10733180; doi:10.1093/ofid/ofad599)
Supplement: ofad599_Supplementary_Data [file ofad599_supplementary_data.docx]

**Supplemental Figure 1. FluSurv-NET Case Report Form: Underlying Condition Categories Section**


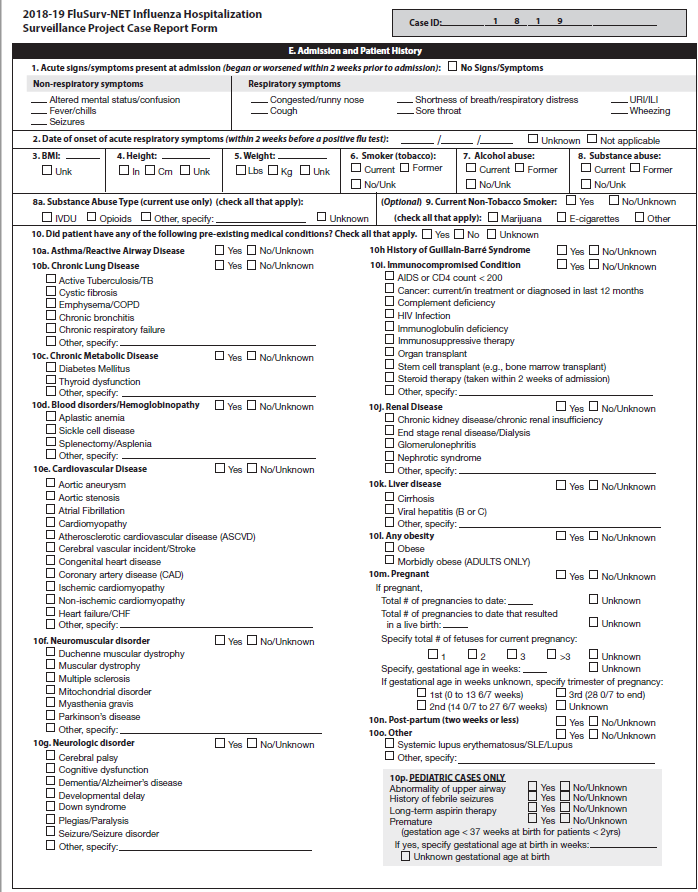


**Supplemental Figure 2. Influenza Vaccination by Age Group, Sex, and Race/Ethnicity among Adults Aged 18-49 years Hospitalized with Influenza, FluSurv-NET, 2011-12—2018-19**


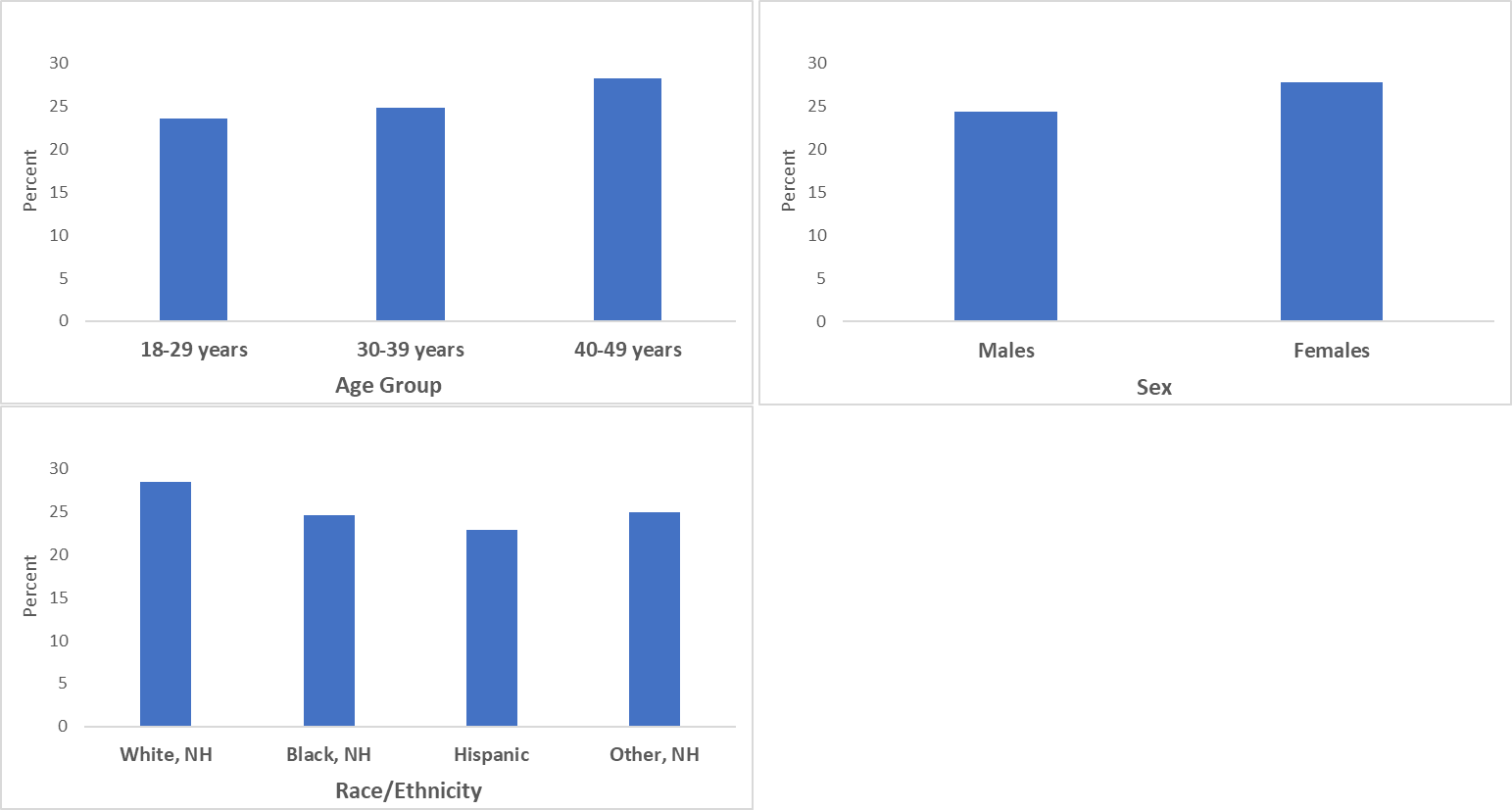


**Supplemental Table 1. Pearson Correlation Coefficients for Underlying Condition Categories among Adults Aged 18-49 years**

**Hospitalized with Influenza, FluSurv-NET, 2011-12—2018-19**

|  | Asthma | Blood Disorders | Chronic Lung Disease | Cardiovascular Disease | Neurologic Disorder | Immuno-compromised | Liver Disease | Morbid Obesity | Chronic Metabolic Disease | Renal Disease |
| --- | --- | --- | --- | --- | --- | --- | --- | --- | --- | --- |
| Asthma | 1.000 | -0.024 | 0.101 | -0.027 | -0.033 | 0.014 | -0.029 | 0.122 | -0.028 | -0.093 |
| Blood Disorders | -0.024 | 1.000 | 0.007 | 0.095 | 0.058 | 0.039 | 0.037 | -0.033 | -0.017 | 0.052 |
| Chronic Lung Disease | 0.101 | 0.007 | 1.000 | 0.147 | 0.081 | 0.053 | 0.054 | 0.072 | 0.077 | 0.014 |
| Cardiovascular Disease | -0.027 | 0.095 | 0.147 | 1.000 | 0.115 | 0.057 | 0.026 | 0.081 | 0.147 | 0.241 |
| Neurologic Disorder | -0.033 | 0.058 | 0.081 | 0.115 | 1.000 | -0.020 | 0.022 | -0.050 | 0.021 | 0.025 |
| Immunocompromised | 0.014 | 0.039 | 0.053 | 0.057 | -0.020 | 1.000 | 0.059 | -0.045 | 0.013 | 0.172 |
| Liver Disease | -0.029 | 0.037 | 0.054 | 0.026 | 0.022 | 0.059 | 1.000 | -0.027 | 0.018 | 0.040 |
| Extreme Obesity | 0.122 | -0.033 | 0.072 | 0.081 | -0.050 | -0.045 | -0.027 | 1.000 | 0.130 | -0.007 |
| Chronic Metabolic Disease | -0.028 | -0.017 | 0.077 | 0.147 | 0.021 | 0.013 | 0.018 | 0.130 | 1.000 | 0.184 |
| Renal Disease | -0.093 | 0.052 | 0.014 | 0.241 | 0.025 | 0.172 | 0.040 | -0.007 | 0.184 | 1.000 |

**Supplemental Table 2a. Factors associated with severe outcomes among adults aged 18-49 years hospitalized with influenza, unadjusted analyses, FluSurv-NET, 2011-12—2018-19**

|  | **N** | **ICU Admission ^1^** | | | **Mechanical Ventilation ^1^** | | |
| --- | --- | --- | --- | --- | --- | --- | --- |
|  |  | **Yes** | **No** | **Odds ratio (95% CI)** | **Yes** | **No** | **Odds ratio (95% CI)** |
|  |  | **(n, %)** | **(n, %)** |  | **(n, %)** | **(n, %)** |  |
| All adults 18-49 years old | 16,140 | 3,129 (19.5) | 12,927 (80.5) |  | 1,340 (8.4) | 14,699 (91.6) |  |
| **Age Group** | | | | | | | |
| 18-29 years ^2^ | 3,788 | 710 (18.8) | 3,057 (81.2) | --- | 239 (6.4) | 3,524 (93.6) | --- |
| 30-39 years | 4,706 | 887 (18.9) | 3,798 (81.1) | 1.01 (0.90-1.12) | 387 (8.3) | 4,298 (91.7) | 1.33 (1.12-1.57) |
| 40-49 years | 7,646 | 1,532 (20.1) | 6,072 (79.9) | 1.09 (0.98-1.20) | 714 (9.4) | 6,877 (90.6) | 1.53 (1.32-1.78) |
| **Sex** | | | | | | | |
| Males ^2^ | 7,746 | 1,595 (20.7) | 6,100 (79.3) | --- | 694 (9.0) | 6,999 (91.0) | --- |
| Females | 8,394 | 1,534 (18.3) | 6,827 (81.7) | 0.86 (0.79-0.93) | 646 (7.7) | 7,700 (92.3) | 0.85 (0.76-0.95) |
| **Race/Ethnicity** | | | | | | | |
| White, NH ^2^ | 7,123 | 1,539 (21.7) | 5,537 (78.3) | --- | 676 (9.6) | 6,392 (90.4) | --- |
| Black, NH | 4,930 | 779 (15.8) | 4,137 (84.2) | 0.68 (0.62-0.75) | 312 (6.3) | 4,603 (93.7) | 0.64 (0.56-0.74) |
| Other, NH ^3^ | 2,098 | 404 (19.5) | 1,672 (80.5) | 0.87 (0.77-0.98) | 184 (8.9) | 1,890 (91.1) | 0.92 (0.78-1.09) |
| Hispanic | 1,989 | 407 (20.5) | 1,581 (79.5) | 0.93 (0.82-1.05) | 168 (8.5) | 1,814 (91.5) | 0.88 (0.73-1.05) |
| **Influenza Season** | | | | | | | |
| 2011-2012 ^2^ | 433 | 67 (15.5) | 364 (84.5) | --- | 28 (6.5) | 403 (93.5) | --- |
| 2012-2013 | 1,605 | 280 (17.5) | 1,319 (82.5) | 1.15 (0.86-1.54) | 115 (7.2) | 1,483 (92.8) | 1.12 (0.73-1.71) |
| 2013-2014 | 2,368 | 549 (23.3) | 1,812 (76.7) | 1.65 (1.25-2.17) | 314 (13.3) | 2,043 (86.7) | 2.21 (1.48-3.30) |
| 2014-2015 | 1,908 | 314 (16.6) | 1,573 (83.4) | 1.08 (0.81-1.45) | 127 (6.7) | 1,760 (93.3) | 1.04 (0.68-1.59) |
| 2015-2016 | 1,801 | 360 (20.1) | 1,435 (79.9) | 1.36 (1.02-1.81) | 182 (10.1) | 1,613 (89.9) | 1.62 (1.07-2.45) |
| 2016-2017 | 1,902 | 361 (19.1) | 1,533 (80.9) | 1.28 (0.96-1.70) | 124 (6.6) | 1,768 (93.4) | 1.01 (0.66-1.54) |
| 2017-2018 | 3,337 | 657 (19.8) | 2,666 (80.2) | 1.34 (1.02-1.76) | 228 (6.9) | 3,085 (93.1) | 1.06 (0.71-1.60) |
| 2018-2019 | 2,786 | 541 (19.6) | 2,225 (80.4) | 1.32 (1.00-1.74) | 222 (8.0) | 2,544 (92.0) | 1.26 (0.84-1.89) |
| **Influenza Type** | | | | | | | |
| Influenza A ^2^ | 13,140 | 2,573 (19.7) | 10,500 (80.3) | --- | 1,114 (8.5) | 11,942 (91.5) | --- |
| Influenza A&B | 77 | 17 (22.1) | 60 (77.9) | 1.16 (0.67-1.98) | 9 (11.7) | 68 (88.3) | 1.42 (0.71-2.85) |
| Influenza A/B not distinguished | 26 | 3 (11.5) | 23 (88.5) | 0.53 (0.16-1.77) | 2 (7.7) | 24 (92.3) | 0.89 (0.21-3.78) |
| Influenza B | 2,891 | 533 (18.5) | 2,341 (81.5) | 0.93 (0.84-1.03) | 214 (7.4) | 2,660 (92.6) | 0.86 (0.74-1.00) |
| Unknown | 6 | 3 (50.0) | 3 (50.0) | 4.08 (0.82-20.23) | 1 (16.7) | 5 (83.3) | 2.14 (0.25-18.37) |
| **Influenza vaccination status** | | | | | | | |
| Yes ^2^ | 4,220 | 766 (18.2) | 3,440 (81.8) | --- | 301 (7.2) | 3,904 (92.8) | --- |
| No | 9,453 | 1,832 (19.4) | 7,600 (80.6) | 1.08 (0.99-1.19) | 806 (8.6) | 8,615 (91.4) | 1.21 (1.06-1.39) |
| Unknown | 2,467 | 531 (22.0) | 1,887 (78.0) | 1.26 (1.12-1.43) | 233 (9.7) | 2,180 (90.3) | 1.39 (1.16-1.66) |
| **Antiviral Treatment** | | | | | | | |
| Yes ^2^ | 14,100 | 2,837 (20.2) | 11,230 (79.8) | --- | 1,226 (8.7) | 12,828 (91.3) | --- |
| No | 2,040 | 292 (14.7) | 1,697 (85.3) | 0.68 (0.60-0.78) | 114 (5.7) | 1,871 (94.3) | 0.64 (0.52-0.78) |
| **Symptoms at admission ^4^** | 15,919 | 3,097 (19.5) | 12,790 (80.5) | 1.04 (0.70-1.53) | 1,323 (8.3) | 14,551 (91.7) | 0.79 (0.48-1.31) |
| **Tobacco Smoker** | | | | | | | |
| Current smoker ^2^ | 5,370 | 1,109 (20.7) | 4,252 (79.3) |  | 489 (9.1) | 4,869 (90.9) |  |
| Former smoker | 1,948 | 363 (18.6) | 1,584 (81.4) | 0.88 (0.77-1.00) | 148 (7.6) | 1,798 (92.4) | 0.82 (0.68-0.99) |
| No/Unknown smoker | 8,822 | 1,657 (18.9) | 7,091 (81.1) | 0.90 (0.82-0.98) | 703 (8.0) | 8,032 (92.0) | 0.87 (0.77-0.98) |
| **Underlying Medical Conditions ^5^** | | | | | | | |
| Asthma | 5,157 | 855 (16.6) | 4,296 (83.4) | 0.75 (0.69-0.82) | 331 (6.4) | 4,818 (93.6) | 0.67 (0.59-0.76) |
| Blood disorders | 904 | 151 (16.8) | 750 (83.2) | 0.82 (0.69-0.98) | 78 (8.7) | 823 (91.3) | 1.04 (0.82-1.32) |
| Chronic Lung Disease | 2,093 | 508 (24.3) | 1,583 (75.7) | 1.39 (1.24-1.55) | 231 (11.1) | 1,854 (88.9) | 1.44 (1.24-1.67) |
| Chronic Metabolic Disease | 4,262 | 1,087 (25.6) | 3,165 (74.4) | 1.64 (1.51-1.78) | 391 (9.2) | 3,858 (90.8) | 1.16 (1.02-1.31) |
| Cardiovascular Disease | 2,749 | 653 (23.8) | 2,091 (76.2) | 1.36 (1.24-1.50) | 281 (10.3) | 2,459 (89.7) | 1.32 (1.15-1.52) |
| Coronary artery disease | 527 | 124 (23.5) | 403 (76.5) | 1.28 (1.04-1.57) | 44 (8.4) | 482 (91.6) | 1.00 (0.73-1.37) |
| Diabetes | 3,229 | 860 (26.7) | 2,361 (73.3) | 1.69 (1.55-1.85) | 294 (9.1) | 2,926 (90.9) | 1.13 (0.99-1.29) |
| COPD | 1,188 | 292 (24.6) | 896 (75.4) | 1.38 (1.20-1.58) | 122 (10.3) | 1,061 (89.7) | 1.29 (1.06-1.56) |
| Neurologic disorder | 2,743 | 699 (25.5) | 2,039 (74.5) | 1.53 (1.39-1.69) | 324 (11.9) | 2,408 (88.1) | 1.63 (1.42-1.86) |
| Immunocompromised | 3,101 | 513 (16.6) | 2,579 (83.4) | 0.78 (0.71-0.87) | 218 (7.0) | 2,875 (93.0) | 0.80 (0.69-0.93) |
| Renal Disease | 1,705 | 347 (20.4) | 1,353 (79.6) | 1.06 (0.94-1.21) | 142 (8.4) | 1,558 (91.6) | 1.00 (0.83-1.20) |
| Liver disease | 747 | 184 (24.7) | 562 (75.3) | 1.37 (1.16-1.63) | 92 (12.4) | 652 (87.6) | 1.59 (1.27-1.99) |
| Thyroid dysfunction | 1,194 | 281 (23.6) | 911 (76.4) | 1.30 (1.13-1.49) | 113 (9.5) | 1,077 (90.5) | 1.16 (0.95-1.42) |
| Sickle cell disease | 325 | 19 (5.9) | 304 (94.1) | 0.25 (0.16-0.40) | 5 (1.5) | 319 (98.5) | 0.17 (0.07-0.41) |
| HIV Infection | 740 | 103 (14.0) | 632 (86.0) | 0.66 (0.53-0.82) | 41 (5.6) | 695 (94.4) | 0.63 (0.46-0.87) |
| Obesity and extreme Obesity | 7,083 | 1,347 (19.0) | 5,725 (81.0) | 0.95 (0.87-1.02) | 643 (9.1) | 6,420 (90.9) | 1.18 (1.06-1.33) |
| Extreme Obesity | 2,653 | 554 (20.9) | 2,097 (79.1) | 1.11 (1.00-1.23) | 262 (9.9) | 2,384 (90.1) | 1.25 (1.09-1.45) |
| No Underlying Conditions | 2,490 | 383 (15.4) | 2,103 (84.6) | 0.72 (0.64-0.80) | 180 (7.2) | 2,305 (92.8) | 0.83 (0.71-0.98) |

^1^ Cases with unknown and missing for outcomes variables were excluded. For ICU admission, 34 (0.2%) cases were missing and 50 (0.3%) cases were unknown. For Invasive mechanical ventilation, 43 (0.3%) cases were missing and 58 (0.4%) cases were unknown. For in-hospital death, 35 (0.2%) cases were missing and 18 (0.1%) cases were unknown. For Pneumonia, no cases were missing or unknown. Pneumonia was defined as a combination of radiographic findings of bronchopneumonia, air space opacity, consolidation, lobar or interstitial infiltrate within 3 days of hospital admission, and either an International Classification of Diseases–coded discharge diagnosis of pneumonia or documentation of pneumonia on the hospital discharge summary.

^2^ Reference group for odds ratio (95% C.I.) and p-values comparing groups within categories.

^3^ "Other, NH" Race/Ethnicity includes American Indian/Alaska Native (n=174), Asian Pacific Islander (n=615), Multiracial (n=63), unknown (n=1234), or missing (n=12)

^4^ Any symptoms at admission include Acute respiratory illness, Asthma and/or COPD exacerbation, Pneumonia, Other respiratory or cardiac conditions (seasons 2011-2012 - 2013-2014); Chest pain, Conjunctivitis/pink eye, Diarrhea, Headache, Myalgia/muscle aches, Nausea/vomiting, Rash (seasons 2014-2015 - 2017-2018); Altered mental status/confusion, Congested/runny nose, Cough, Seizures, Shortness of breath/respiratory distress, Sore throat, Wheezing, URI/ILI (seasons 2014-2015 - 2018-2019); Fatigue/weakness (seasons 2015-2016 - 2017-2018); Fever/chills (seasons 2012-2013 - 2018-2019); and Other, non-respiratory symptoms (seasons 2011-2012 - 2017-2018). 111 (0.7%) cases are missing data for all symptoms.

^5^ Reference group for each Underlying Medical condition is the absence of the condition. Data on underlying conditions missing for n=246 (1.5%) cases . **Supplemental Table 2b. Factors associated with in-hospital death and pneumonia among aged adults 18-49 years hospitalized with influenza, unadjusted analyses, FluSurv-NET, 2011-12—2018-19**

|  | **In-Hospital Death ^1^** | | | **Pneumonia ^1^** | | |
| --- | --- | --- | --- | --- | --- | --- |
|  | **Yes** | **No** | **Odds ratio (95% CI)** | **Yes** | **No** | **Odds ratio (95% CI)** |
|  | **(n, %)** | **(n, %)** |  | **(n, %)** | **(n, %)** |  |
| All adults 18-49 years old | 269 (1.7) | 15,818 (98.3) |  | 3,893 (24.1) | 12,247 (75.9) |  |
| **Age Group** | | | | | | |
| 18-29 years ^2^ | 38 (1.0) | 3,733 (99.0) | --- | 708 (18.7) | 3,080 (81.3) | --- |
| 30-39 years | 69 (1.5) | 4,624 (98.5) | 1.47 (0.98-2.18) | 1,195 (25.4) | 3,511 (74.6) | 1.48 (1.33-1.64) |
| 40-49 years | 162 (2.1) | 7,461 (97.9) | 2.13 (1.49-3.04) | 1,990 (26.0) | 5,656 (74.0) | 1.53 (1.39-1.69) |
| **Sex** | | | | | | |
| Males ^2^ | 149 (1.9) | 7,570 (98.1) | --- | 2,018 (26.1) | 5,728 (73.9) | --- |
| Females | 120 (1.4) | 8,248 (98.6) | 0.74 (0.58-0.94) | 1,875 (22.3) | 6,519 (77.7) | 0.82 (0.76-0.88) |
| **Race/Ethnicity** | | | | | | |
| White, NH ^2^ | 153 (2.2) | 6,942 (97.8) | --- | 1,831 (25.7) | 5,292 (74.3) | --- |
| Black, NH | 46 (0.9) | 4,879 (99.1) | 0.43 (0.31-0.60) | 1,015 (20.6) | 3,915 (79.4) | 0.75 (0.69-0.82) |
| Other, NH ^3^ | 34 (1.6) | 2,045 (98.4) | 0.75 (0.52-1.10) | 529 (25.2) | 1,569 (74.8) | 0.97 (0.87-1.09) |
| Hispanic | 36 (1.8) | 1,952 (98.2) | 0.84 (0.58-1.21) | 518 (26.0) | 1,471 (74.0) | 1.02 (0.91-1.14) |
| **Influenza Season** | | | | | | |
| 2011-2012 * | 6 (1.4) | 426 (98.6) | --- | 93 (21.5) | 340 (78.5) | --- |
| 2012-2013 | 21 (1.3) | 1,579 (98.7) | 0.94 (0.38-2.35) | 323 (20.1) | 1,282 (79.9) | 0.92 (0.71-1.19) |
| 2013-2014 | 66 (2.8) | 2,298 (97.2) | 2.04 (0.88-4.73) | 734 (31.0) | 1,634 (69.0) | 1.64 (1.28-2.10) |
| 2014-2015 | 18 (1.0) | 1,874 (99.0) | 0.68 (0.27-1.73) | 347 (18.2) | 1,561 (81.8) | 0.81 (0.63-1.05) |
| 2015-2016 | 32 (1.8) | 1,767 (98.2) | 1.29 (0.53-3.09) | 554 (30.8) | 1,247 (69.2) | 1.62 (1.26-2.09) |
| 2016-2017 | 29 (1.5) | 1,869 (98.5) | 1.10 (0.45-2.67) | 389 (20.5) | 1,513 (79.5) | 0.94 (0.73-1.21) |
| 2017-2018 | 54 (1.6) | 3,275 (98.4) | 1.17 (0.50-2.74) | 740 (22.2) | 2,597 (77.8) | 1.04 (0.82-1.33) |
| 2018-2019 | 43 (1.6) | 2,730 (98.4) | 1.12 (0.47-2.64) | 713 (25.6) | 2,073 (74.4) | 1.26 (0.98-1.61) |
| **Influenza Type** | | | | | | |
| Influenza A ^2^ | 213 (1.6) | 12,881 (98.4) | --- | 3,203 (24.4) | 9,937 (75.6) | --- |
| Influenza A&B | 4 (5.2) | 73 (94.8) | 3.31 (1.20-9.15) | 22 (28.6) | 55 (71.4) | 1.24 (0.76-2.04) |
| Influenza A/B not distinguished | 1 (3.8) | 25 (96.2) | 2.42 (0.33-17.93) | 8 (30.8) | 18 (69.2) | 1.38 (0.60-3.17) |
| Influenza B | 50 (1.7) | 2,834 (98.3) | 1.07 (0.78-1.46) | 659 (22.8) | 2,232 (77.2) | 0.92 (0.83-1.01) |
| Unknown | 1 (16.7) | 5 (83.3) | 12.09 (1.41-103.97) | 1 (16.7) | 5 (83.3) | 0.62 (0.07-5.31) |
| **Influenza vaccination status** | | | | | | |
| Yes ^2^ | 53 (1.3) | 4,165 (98.7) | --- | 876 (20.8) | 3,344 (79.2) | --- |
| No | 153 (1.6) | 9,289 (98.4) | 1.29 (0.95-1.77) | 2,425 (25.7) | 7,028 (74.3) | 1.32 (1.21-1.44) |
| Unknown | 63 (2.6) | 2,364 (97.4) | 2.09 (1.45-3.03) | 592 (24.0) | 1,875 (76.0) | 1.21 (1.07-1.36) |
| **Antiviral Treatment** | | | | | | |
| Yes ^2^ | 227 (1.6) | 13,861 (98.4) | --- | 3,484 (24.7) | 10,616 (75.3) | --- |
| No | 42 (2.1) | 1,957 (97.9) | 1.31 (0.94-1.83) | 409 (20.0) | 1,631 (80.0) | 0.76 (0.68-0.86) |
| **Symptoms at admission ^4^** | 255 (1.6) | 15,654 (98.4) | 0.19 (0.11-0.33) | 3,870 (24.3) | 12,049 (75.7) | 2.77 (1.79-4.26) |
| **Tobacco Smoker** | | | | | | |
| Current smoker^2^ | 79 (1.5) | 5,288 (98.5) | --- | 1,387 (25.8) | 3,983 (74.2) | --- |
| Former smoker | 38 (2.0) | 1,908 (98.0) | 1.33 (0.90-1.97) | 427 (21.9) | 1,521 (78.1) | 0.81 (0.71-0.91) |
| No/Unknown | 152 (1.7) | 8,622 (98.3) | 1.18 (0.90-1.55) | 2,079 (23.6) | 6,743 (76.4) | 0.89 (0.82-0.96) |
| **Underlying Medical Conditions ^5^** | | | | | | |
| Asthma | 34 (0.7) | 5,119 (99.3) | 0.30 (0.21-0.43) | 948 (18.4) | 4,209 (81.6) | 0.61 (0.56-0.66) |
| Blood disorders | 17 (1.9) | 887 (98.1) | 1.14 (0.69-1.87) | 194 (21.5) | 710 (78.5) | 0.85 (0.72-1.00) |
| Chronic Lung Disease | 42 (2.0) | 2,050 (98.0) | 1.25 (0.89-1.74) | 559 (26.7) | 1,534 (73.3) | 1.16 (1.05-1.29) |
| Chronic Metabolic Disease | 89 (2.1) | 4,169 (97.9) | 1.39 (1.07-1.80) | 952 (22.3) | 3,310 (77.7) | 0.87 (0.80-0.94) |
| Cardiovascular Disease | 63 (2.3) | 2,686 (97.7) | 1.50 (1.13-2.00) | 671 (24.4) | 2,078 (75.6) | 1.01 (0.92-1.11) |
| Coronary artery disease | 10 (1.9) | 517 (98.1) | 1.15 (0.61-2.17) | 126 (23.9) | 401 (76.1) | 0.98 (0.80-1.20) |
| Diabetes | 67 (2.1) | 3,160 (97.9) | 1.33 (1.01-1.76) | 699 (21.6) | 2,530 (78.4) | 0.83 (0.76-0.91) |
| COPD | 21 (1.8) | 1,167 (98.2) | 1.07 (0.68-1.67) | 288 (24.2) | 900 (75.8) | 1.00 (0.87-1.15) |
| Neurologic disorder | 66 (2.4) | 2,675 (97.6) | 1.60 (1.21-2.13) | 731 (26.6) | 2,012 (73.4) | 1.17 (1.06-1.28) |
| Immunocompromised | 56 (1.8) | 3,042 (98.2) | 1.11 (0.82-1.49) | 649 (20.9) | 2,452 (79.1) | 0.79 (0.72-0.87) |
| Renal Disease | 36 (2.1) | 1,669 (97.9) | 1.31 (0.92-1.87) | 380 (22.3) | 1,325 (77.7) | 0.88 (0.78-1.00) |
| Liver disease | 31 (4.1) | 716 (95.9) | 2.76 (1.88-4.04) | 186 (24.9) | 561 (75.1) | 1.04 (0.88-1.23) |
| Thyroid dysfunction | 25 (2.1) | 1,168 (97.9) | 1.29 (0.85-1.95) | 265 (22.2) | 929 (77.8) | 0.88 (0.77-1.02) |
| Sickle cell disease | 1 (0.3) | 324 (99.7) | 0.18 (0.03-1.28) | 37 (11.4) | 288 (88.6) | 0.40 (0.28-0.56) |
| HIV Infection | 9 (1.2) | 730 (98.8) | 0.72 (0.37-1.40) | 194 (26.2) | 546 (73.8) | 1.12 (0.94-1.32) |
| Obesity and extreme Obesity | 121 (1.7) | 6,955 (98.3) | 1.05 (0.82-1.34) | 1,736 (24.5) | 5,347 (75.5) | 1.02 (0.95-1.10) |
| Extreme Obesity | 56 (2.1) | 2,596 (97.9) | 1.34 (1.00-1.81) | 674 (25.4) | 1,979 (74.6) | 1.08 (0.98-1.19) |
| No Underlying Conditions | 34 (1.4) | 2,454 (98.6) | 0.79 (0.55-1.13) | 752 (30.2) | 1,738 (69.8) | 1.44 (1.31-1.58) |

^1^ Cases with unknown and missing for outcomes variables were excluded. For ICU admission, 34 (0.2%) cases were missing and 50 (0.3%) cases were unknown. For Invasive mechanical ventilation, 43 (0.3%) cases were missing and 58 (0.4%) cases were unknown. For in-hospital death, 35 (0.2%) cases were missing and 18 (0.1%) cases were unknown. Pneumonia was defined as a combination of radiographic findings of bronchopneumonia, air space opacity, consolidation, lobar or interstitial infiltrate within 3 days of hospital admission, and either an International Classification of Diseases–coded discharge diagnosis of pneumonia or documentation of pneumonia on the hospital discharge summary.

^2^ Reference group for odds ratio (95% C.I.) and p-values comparing groups within categories

^3^ "Other, NH" Race/Ethnicity includes American Indian/Alaska Native (n=174), Asian Pacific Islander (n=615), Multiracial (n=63), unknown (n=1234), or missing (n=12)

^4^ Any symptoms at admission include Acute respiratory illness, Asthma and/or COPD exacerbation, Pneumonia, Other respiratory or cardiac conditions (seasons 2011-2012 - 2013-2014); Chest pain, Conjunctivitis/pink eye, Diarrhea, Headache, Myalgia/muscle aches, Nausea/vomiting, Rash (seasons 2014-2015 - 2017-2018); Altered mental status/confusion, Congested/runny nose, Cough, Seizures, Shortness of breath/respiratory distress, Sore throat, Wheezing, URI/ILI (seasons 2014-2015 - 2018-2019); Fatigue/weakness (seasons 2015-2016 - 2017-2018); Fever/chills (seasons 2012-2013 - 2018-2019); and Other, non-respiratory symptoms (seasons 2011-2012 - 2017-2018). 111 (0.7%) cases are missing data for all symptoms.

^5^ Reference group for each Underlying Medical condition is the absence of the condition. Data on underlying conditions missing for n=246 (1.5%) cases

**Supplemental Table 3. Multivariable logistic regression^1^ analysis of factors associated with severe outcomes among adults aged 18-49 years hospitalized with influenza, FluSurv-NET, 2011-12—2018-19**

| **Variable** | **Pneumonia^2^** | **ICU Admission^2^** | **Invasive mechanical ventilation^2^** | **In-Hospital Death^2^** |
| --- | --- | --- | --- | --- |
|  | **aOR (95% CI)** | **aOR (95% CI)** | **aOR (95% CI)** | **aOR (95% CI)** |
| **Age Group** |  |  |  |  |
| 30-39 vs 18-29 years | 1.44 (1.29-1.61) | 0.91 (0.82-1.02) | 1.25 (1.05-1.48) | 1.28 (0.86-1.94) |
| 40-49 vs 18-29 years | 1.51 (1.36-1.67) | 0.9 (0.81-1) | 1.36 (1.16-1.59) | 1.69 (1.18-2.48) |
| **Sex** |  |  |  |  |
| Female vs Male | 0.89 (0.83-0.96) | 0.9 (0.83-0.98) | 0.91 (0.81-1.02) | 0.85 (0.66-1.09) |
| **Race/Ethnicity** |  |  |  |  |
| Black, NH vs White, NH | 0.88 (0.8-0.97) | 0.75 (0.68-0.84) | 0.72 (0.62-0.84) | 0.47 (0.32-0.66) |
| Hispanic vs White, NH | 1.02 (0.9-1.15) | 0.95 (0.83-1.09) | 0.98 (0.81-1.18) | 0.98 (0.65-1.45) |
| Other^3^, NH vs White, NH | 0.99 (0.87-1.11) | 0.88 (0.77-1) | 0.98 (0.81-1.17) | 0.82 (0.54-1.2) |
| **Flu vaccination status** |  |  |  |  |
| Unvaccinated vs Vaccinated | 1.23 (1.12-1.36) | 1.18 (1.07-1.3) | 1.25 (1.08-1.45) | 1.48 (1.07-2.09) |
| Unknown vs Vaccinated | 1.17 (1.03-1.33) | 1.4 (1.23-1.6) | 1.54 (1.27-1.86) | 2.43 (1.64-3.61) |
| **Tobacco use** |  |  |  |  |
| Current Smoker vs Non-smoker | 1.07 (0.98-1.16) | 1.1 (1.01-1.21) | 1.03 (0.91-1.18) | 0.74 (0.55-0.99) |
| Former Smoker vs Non-smoker | 0.9 (0.79-1.01) | 0.96 (0.84-1.09) | 0.87 (0.72-1.05) | 1.02 (0.69-1.47) |
| **Medical Conditions** |  |  |  |  |
| Asthma | 0.67 (0.61-0.73) | 0.76 (0.69-0.84) | 0.68 (0.59-0.79) | 0.34 (0.23-0.49) |
| Blood disorders | 0.96 (0.81-1.14) | 0.82 (0.68-0.99) | 1.02 (0.79-1.3) | 1.1 (0.63-1.81) |
| Chronic Lung Disease | 1.25 (1.12-1.41) | 1.28 (1.14-1.44) | 1.34 (1.14-1.58) | 1.1 (0.76-1.57) |
| Cardiovascular Disease | 1.03 (0.93-1.14) | 1.23 (1.1-1.37) | 1.17 (1.01-1.36) | 1.29 (0.94-1.75) |
| Chronic Metabolic Disease | 0.86 (0.78-0.94) | 1.56 (1.42-1.71) | 1.05 (0.92-1.2) | 1.2 (0.91-1.58) |
| Neurologic Disorder | 1.28 (1.15-1.42) | 1.44 (1.3-1.6) | 1.54 (1.33-1.78) | 1.53 (1.12-2.06) |
| Immunocompromising Condition | 0.91 (0.82-1.01) | 0.81 (0.72-0.9) | 0.83 (0.71-0.98) | 1.19 (0.86-1.62) |
| Liver Disease | 0.96 (0.8-1.14) | 1.22 (1.02-1.46) | 1.38 (1.08-1.74) | 2.36 (1.55-3.48) |
| Extreme Obesity | 1.13 (1.02-1.26) | 1.04 (0.93-1.16) | 1.23 (1.06-1.43) | 1.43 (1.04-1.95) |
| No Underlying Medical Conditions | 1.19 (1.06-1.34) | 0.76 (0.66-0.87) | 0.82 (0.68-1) | 0.8 (0.52-1.21) |

1 Each model was also adjusted for influenza season and surveillance site

2 Cases with unknown and missing for outcomes variables were excluded. For ICU admission, 34 (0.2%) cases were missing and 50 (0.3%) cases were unknown. For Invasive mechanical ventilation, 43 (0.3%) cases were missing and 58 (0.4%) cases were unknown. For in-hospital death, 35 (0.2%) cases were missing and 18 (0.1%) cases were unknown. For Pneumonia, no cases were missing or unknown. Pneumonia was defined as a combination of radiographic findings of bronchopneumonia, air space opacity, consolidation, lobar or interstitial infiltrate within 3 days of hospital admission, and either an International Classification of Diseases–coded discharge diagnosis of pneumonia or documentation of pneumonia on the hospital discharge summary.

3 "Other, NH" Race/Ethnicity includes American Indian/Alaska Native (n=174), Asian Pacific Islander (n=615), Multiracial (n=63), unknown (n=1234), or missing (n=12)

**Supplemental Table 4. Multivariable logistic regression^1^ analysis of the association between number of underlying condition categories and severe outcomes among adults aged 18-49 years hospitalized with influenza, FluSurv-NET, 2011-12—2018-19**

| **Effect** | **Pneumonia^2^** | **ICU Admission^2^** | **Invasive mechanical ventilation^2^** | **In-Hospital Death^2^** |
| --- | --- | --- | --- | --- |
|  | **aOR (95% CI)** | **aOR (95% CI)** | **aOR (95% CI)** | **aOR (95% CI)** |
| **Age Group** |  |  |  |  |
| 30-39 vs 18-29 | 1.46 (1.31-1.62) | 0.95 (0.85-1.06) | 1.26 (1.07-1.5) | 1.37 (0.92-2.06) |
| 40-49 vs 18-29 | 1.55 (1.4-1.71) | 0.99 (0.89-1.1) | 1.41 (1.21-1.65) | 1.85 (1.3-2.7) |
| **Sex** |  |  |  |  |
| Female vs Male | 0.85 (0.79-0.91) | 0.87 (0.8-0.94) | 0.87 (0.77-0.97) | 0.76 (0.59-0.97) |
| **Race/Ethnicity** |  |  |  |  |
| Black, NH vs White, NH | 0.84 (0.76-0.92) | 0.7 (0.63-0.78) | 0.67 (0.58-0.78) | 0.42 (0.29-0.6) |
| Hispanic vs White, NH | 1 (0.88-1.12) | 0.94 (0.83-1.07) | 0.95 (0.78-1.14) | 0.96 (0.64-1.41) |
| Other^3^, NH vs White, NH | 0.96 (0.85-1.08) | 0.86 (0.76-0.98) | 0.95 (0.79-1.13) | 0.83 (0.55-1.22) |
| **Influenza vaccination status** |  |  |  |  |
| Unvaccinated vs Vaccinated | 1.2 (1.09-1.32) | 1.13 (1.02-1.24) | 1.22 (1.06-1.41) | 1.45 (1.06-2.03) |
| Unknown vs Vaccinated | 1.15 (1.02-1.3) | 1.34 (1.18-1.53) | 1.5 (1.25-1.81) | 2.43 (1.65-3.58) |
| **Tobacco use** |  |  |  |  |
| Current Smoker vs Non-smoker | 1.05 (0.96-1.14) | 1.09 (0.99-1.19) | 1.04 (0.91-1.17) | 0.74 (0.55-0.97) |
| Former Smoker vs Non-smoker | 0.87 (0.77-0.98) | 0.94 (0.82-1.07) | 0.84 (0.69-1.01) | 0.97 (0.66-1.38) |
| **Medical Conditions** |  |  |  |  |
| 0 vs 1 | 0.87 (0.76-0.99) | 1.2 (1.02-1.41) | 1.22 (0.97-1.53) | 1.07 (0.63-1.8) |
| 0 vs 2 | 0.68 (0.59-0.77) | 1.49 (1.29-1.72) | 1.04 (0.84-1.3) | 0.93 (0.56-1.53) |
| 0 vs 3+ | 0.76 (0.68-0.84) | 1.68 (1.48-1.9) | 1.45 (1.22-1.73) | 1.74 (1.2-2.6) |

1 Each model was also adjusted for influenza season and surveillance site

2 Cases with unknown and missing for outcomes variables were recoded as ""No"". For ICU admission, 34 (0.2%) cases were missing and 50 (0.3%) cases were unknown. For Invasive mechanical ventilation, 43 (0.3%) cases were missing and 58 (0.4%) cases were unknown. For in-hospital death, 35 (0.2%) cases were missing and 18 (0.1%) cases were unknown. For Pneumonia, no cases were missing or unknown. Pneumonia was defined as a combination of radiographic findings of bronchopneumonia, air space opacity, consolidation, lobar or interstitial infiltrate within 3 days of hospital admission, and either an International Classification of Diseases–coded discharge diagnosis of pneumonia or documentation of pneumonia on the hospital discharge summary.

3 "Other, NH" Race/Ethnicity includes American Indian/Alaska Native (n=174), Asian Pacific Islander (n=615), Multiracial (n=63), unknown (n=1234), or missing (n=12)
